# Integrated analysis of abnormal metabolic homeostasis for decoding tumor microenvironment

**DOI:** 10.3389/fmolb.2024.1443642

**Published:** 2024-10-15

**Authors:** Qiang Yang, Ying Cai, Shi Qiu, Aihua Zhang

**Affiliations:** ^1^ GAP Research Center and Graduate School, Heilongjiang University of Chinese Medicine, Harbin, China; ^2^ International Advanced Functional Omics Platform, Scientific Experiment Center, Hainan Engineering Research Center for Biological Sample Resources of Major Diseases (First Affiliated Hospital of Hainan Medical University), Key Laboratory of Tropical Cardiovascular Diseases Research of Hainan Province, Hainan Medical University, Haikou, China; ^3^ INTI International University, Nilai, Malaysia

**Keywords:** tumor, metabolism, metabolites, immune escape, tumor microenvironment

**FIGURE 1:**
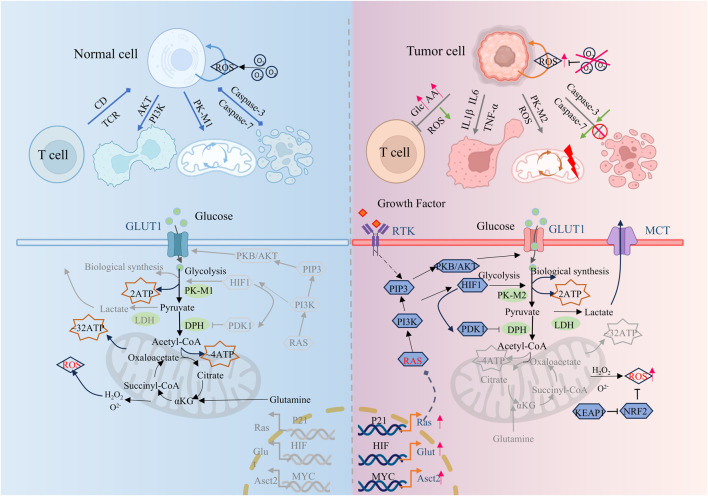
Comparison of metabolic differences between tumor cells and normal cells. Compared to normal cells, tumor cells exhibit tumor-promoting inflammation, enabling replicative immortality, avoiding immune destruction, deregulating cellular energy, resisting cell death, and other characteristics. This figure is created by BioRender.

Tumor cells have physiological characteristics such as malignant proliferation, abnormal differentiation, tissue invasion, and metastasis, which limit the effective treatment of tumors. Although the metabolic states of different types of tumor cells are different, multiple studies have shown that tumor cells are usually accompanied by fundamental metabolic disorders such as energy metabolism, glucose metabolism, lipid metabolism, protein metabolism, and serine metabolism. Tumor cells, with higher than average bioenergy and biosynthetic needs, often maintain their proliferation and survival by efficiently regulating metabolic pathways and fluxes, and effectively combating oxidative stress. (Figure 1). Abnormal metabolism of tumor cells modulates the tumor cell microenvironment by initiating a series of feedback mechanisms that are designed to sustain proliferation and promote tumor cell adaptation. Metabolic abnormalities are ubiquitous in tumor cells and drive tumor occurrence and development. It is of pivotal clinical value to deeply explore and decipher the key localization of metabolic abnormalities in tumor cells, and formulate new tumor prevention and control strategies based on relevant metabolic regulation.

In 2023 and 2024, Nature published a series of crucial roles of metabolic abnormalities in tumor occurrence, development, and treatment, based on which prognosis and treatment were performed. These studies show that abnormal metabolism of tumor cells is a key factor in malignant proliferation and immune escape, and targeting and regulating abnormal metabolism of tumor cells may become a new strategy for tumor treatment. Serine metabolism, as a key driver of tumor occurrence and development, can provide the material and energy basis for tumor cell proliferation, reduce oxidative stress, and promote tumor cell survival (Papalazarou et al., 2023). Some researchers have focused on transmembrane peptides in the serine metabolism pathway in liver cancer cells, and found that transmembrane peptides can competitively inhibit the rate-limiting enzyme of serine synthesis reaction, reduce its enzyme activity, inhibit serine metabolism, and promote the accumulation of intracellular ROS, thereby showing an inhibitory effect on liver cancer cells. Therefore, a new way of treating liver cancer is found in abnormal serine metabolism of liver cancer cells (Wang et al., 2023a). Some researchers have focused on the regulation and metabolism of protein translation in glioblastoma. They found that threonine can catalyze the formation of t^6^A modification by YRDC, which triggers proteome reprogramming, thereby promoting mitotic gene expression. In brief, limiting threonine intake may be a key entry point to limit glioblastoma growth (Wu et al., 2024). Metabolomics, transcriptomics, and comprehensive analysis methods have been widely used to identify potential metabolic biomarkers in tumor cells, focusing on upstream and downstream macromolecular metabolites such as proteins, nucleotides, and lipids. Many metabolic pathways in tumor cells, including fatty acid metabolism, cholesterol metabolism, glutamine metabolism, serine metabolism, carbon unit metabolism, choline metabolism, were reprogrammed. Gong et al. (2023) found that the development and activation of regulatory T cells in nasopharyngeal carcinoma cells is regulated by cell-to-cell interactions mediated by oxidative phosphorylation driven by lipid signaling networks. In summary, mitochondrial integrity, cholesterol accumulation, and fatty acid metabolism in tumor cells work together to improve Tregs function. These studies confirmed that tumor cells can induce immune escape by secreting small molecule metabolites and releasing a variety of immunosuppressive factors to form an immunosuppressive microenvironment. For example, pancreatic cancer-associated fibroblasts can enhance the environmental adaptability of cancer cells by secreting acetic acid acting on the ACSS2-SP1-SAT1 axis (Murthy et al., 2024).

Tumor cells can secrete related metabolites, which act on the tumor microenvironment and affect host function. In a new study, Wang G and other researchers found that tumor cells can release soluble factors into the blood, disrupt the body’s homeostasis, and produce pathological changes in the liver, making it enter an inflammatory state, inducing the secretion of proinflammatory cytokines such as TNF and IL-1α, inhibiting the related lipid metabolic pathways, leading to fat accumulation and liver function damage. This study demonstrates the critical role of multi-tissue cooperation in cancer and helps unravel the mechanism of tumor cell survival (Wang et al., 2023b). It is worth noting that the recovery of abnormal metabolic state by regulating the metabolites related to tumor cells can be used as an important strategy to regulate the efficacy of tumor therapy. Lipid metabolism disorder, as a key factor causing the elevation of aerobic glycolysis levels and the disruption of the tricarboxylic acid cycle in tumor cells, is involved in the synthesis of fatty acids and cholesterol compounds in tumor cells. Studies have found that CD40 drives proinflammatory and anti-tumor polarization in macrophages by regulating fatty acid oxidation and glutamine metabolism. Uncovering new dimensions of immune metabolic regulation and strategies that can restore effective anti-cancer immune responses and help cancer patients reprogram depleted T cells (Liu et al., 2023). Abnormal lipid metabolism in tumor cells is mainly manifested as increased lipid uptake, fatty acid oxidation and storage, etc. These metabolic changes will favor malignant tumor proliferation and tumor microenvironment remodeling.

Due to mutations in tumor genes and the surrounding microenvironment, metabolic processes in tumor cells undergo modifications to support rapid cell growth. The metabolism of tumor cells is subject to reprogramming of their metabolic pathways, and changes in metabolic phenotypes occur as the tumor develops: from nutrient uptake during the early stages of pre-cancerous lesions to altered metabolic subtypes during the local invasion, then to metabolic dependence during tumor metastasis. The metabolism of tumors can effectively promote the proliferation, growth, migration, invasion, and other crucial biological processes in tumor cells. By focusing on tumor cell metabolism and inhibiting their metabolic pathways, the occurrence, and progression of tumors can be effectively prevented (Cai et al., 2023). Therefore, targeting tumor metabolism has become a promising therapeutic and prognostic strategy by taking advantage of the metabolic differences between tumor cells and normal cells, focusing on metabolites, metabolic enzymes, metabolic pathways, etc. Metabolic reprogramming is considered to be an important marker of tumor cells. Abnormal cell metabolism is a key feature of tumorigenesis and development. It is worth noting that drugs targeting the regulation of tumor cell metabolism are still in clinical trials. However, different tumor cells also exhibit unique metabolic characteristics, and the corresponding metabolic treatment strategies need to be developed. To solve these problems, the intersection and extensive cooperation of multiple disciplines are needed (Qiu et al., 2023). The development of cancer treatment strategies from a metabolic perspective requires, on the one hand, the use of molecular biology, metabolomics, genetics and other related research achievements in the field of tumor metabolism. On the other hand, there is a need to combine clinical, epidemiological, molecular imaging and other tumor research experience through a close combination of basic and clinical research. This may hold the promise of uncovering the molecular mechanism of tumor metabolism and assisting in the early intervention and treatment of tumors.
